# Behavior of two *Tannerella forsythia* strains and their cell surface mutants in multispecies oral biofilms

**DOI:** 10.1111/omi.12182

**Published:** 2017-05-22

**Authors:** Susanne Bloch, Thomas Thurnheer, Yukitaka Murakami, Georgios N. Belibasakis, Christina Schäffer

**Affiliations:** ^1^ Department of NanoBiotechnology *NanoGlycobiology* unit Universität für Bodenkultur Vienna Vienna Austria; ^2^ Division of Oral Microbiology and Immunology Institute of Oral Biology Center of Dental Medicine University of Zürich Zürich Switzerland; ^3^ Department of Oral Microbiology Asahi University School of Dentistry Mizuho Gifu Japan; ^4^ Division of Cariology and Endodontics Department of Dental Medicine Karolinska Institute Huddinge Sweden

**Keywords:** *Campylobacter rectus*, cell surface, oral biofilm, periodontal disease, S‐layer glycosylation, *Tannerella forsythia*

## Abstract

As a member of subgingival multispecies biofilms, *Tannerella forsythia* is commonly associated with periodontitis. The bacterium has a characteristic cell surface (S‐) layer modified with a unique *O*‐glycan. Both the S‐layer and the *O*‐glycan were analyzed in this study for their role in biofilm formation by employing an *in vitro* multispecies biofilm model mimicking the situation in the oral cavity. Different *T. forsythia* strains and mutants with characterized defects in cell surface composition were incorporated into the model, together with nine species of select oral bacteria. The influence of the *T. forsythia* S‐layer and attached glycan on the bacterial composition of the biofilms was analyzed quantitatively using colony‐forming unit counts and quantitative real‐time polymerase chain reaction, as well as qualitatively by fluorescence *in situ* hybridization and confocal laser scanning microscopy. This revealed that changes in the *T. forsythia* cell surface did not affect the quantitative composition of the multispecies consortium, with the exception of *Campylobacter rectus* cell numbers. The localization of *T. forsythia* within the bacterial agglomeration varied depending on changes in the S‐layer glycan, and this also affected its aggregation with *Porphyromonas gingivalis*. This suggests a selective role for the glycosylated *T. forsythia* S‐layer in the positioning of this species within the biofilm, its co‐localization with *P. gingivalis*, and the prevalence of *C. rectus*. These findings might translate into a potential role of *T. forsythia* cell surface structures in the virulence of this species when interacting with host tissues and the immune system, from within or beyond the biofilm.

## 1 INTRODUCTION

To proliferate and persist in their habitat, bacteria tend to live predominately in biofilms, which are highly complex and dynamic, polymicrobial communities providing protection from shear forces and host immune responses.^1^ In the oral cavity, multispecies biofilms constitute what is known as “dental plaque”.^2^ In a healthy individual, the oral bacteria exist in a natural balance with their host. However, different factors such as smoking, diabetes, genetic predisposition, or poor dental hygiene can cause the community to become dysbiotic,^3^, ^4^ enabling potentially pathogenic bacteria to increase in numbers and cause persistent infections, such as periodontitis.

It has been recognized that periodontitis has a polymicrobial biofilm etiology and is primarily characterized by a shift in the microbial composition and promotion of growth of Gram‐negative anaerobes; among these are the periodontal pathogens *Porphyromonas gingivalis*,* Treponema denticola*, and *Tannerella forsythia*.^5^ These so‐called “red complex” bacteria are able to subvert host immune responses, modulate the infection process within the subgingival pocket, and promote dysbiosis through the expression of virulence factors.^6^ In the case of *P. gingivalis*, interbacterial interaction and adhesion to host cells are facilitated through the production of colonization factors such as hemagglutinins and fimbriae.^7^ The latter also induce the expression of pro‐inflammatory cytokines, such as interleukin‐1 (IL‐1), IL‐6, IL‐8, and tumor necrosis factor‐α (TNF‐α),^8^ stimulating the immune response during infection. *Porphyromonas gingivalis* further possesses a set of specialized cell surface cysteine proteinases, the gingipains. They can modulate the host immune response through T‐cell receptor cleavage,^9^ proteolytic processing of components of the complement system,^10^ activation of protease‐activated receptors, and inactivation of pro‐ and anti‐inflammatory cytokines.^11^, ^12^, ^13^, ^14^ The oral spirochete *T. denticola* is the only motile member of the “red complex” consortium.^15^ Through the expression of flagellar, chemotactic, and proteolytic factors, *T. denticola* is able to penetrate and directly interact with the gingival epithelium and underlying connective tissue.^16^, ^17^ Here, the principal immunogenic surface antigen of *T. denticola*, the major sheath protein Msp, facilitates actin remodeling and reorganization in host cells and thereby impairs neutrophil chemotaxis and phagocytic activity.^18^, ^19^, ^20^ Through the action of a surface‐associated protease dentilisin, *T. denticola* has been shown to modulate host cell immune responses by degradation of IL‐1β, IL‐6, TNF‐α, and monocyte chemoattractant protein 1.^21^, ^22^

Like *T. denticola, T. forsythia* is characterized by its fastidious growth requirements and is, especially through its initial recalcitrance to genetic manipulation, a less characterized member of the “red complex” consortium. It has been shown to express several putative virulence factors;^23^ among them is its characteristic two‐dimensional (2D) crystalline cell surface (S‐) layer.^24^, ^25^ *Tannerella forsythia* is the only member of the “red complex” consortium that possesses an S‐layer fully covering the bacterial cells; this is formed by self‐assembly of the two S‐layer proteins TfsA and TfsB,^25^ both of which are modified by a unique, complex, branched dekasaccharide that is synthesized by the general protein *O*‐glycosylation system of the bacterium^26^ (Table 1). This dekasaccharide is *O*‐glycosidically bound to multiple serine or threonine residues within a D(S/T)(A/I/L/M/T/V) amino acid target motif present on TfsA and TfsB, but also on several other *T. forsythia* proteins.^26^ S‐layer protein glycosylation was shown to be completed in the bacterial periplasm before glycoprotein export via a type IX secretion system^27^, ^28^ followed by anchoring of the glycoproteins in the cell envelope and equimolar self‐assembly into the mature S‐layer lattice at the cell surface. Given the nanometer‐scaled periodicity of the 2D S‐layer lattice, this strategy results in a high‐density cell surface display of *O*‐glycans. This surface glycosylation affects the physicochemical properties of the bacterial cell surface through the introduction of charged sugar residues (for structure of the *O*‐glycan see Table 1) and modulates bacterial cell hydrophobicity. The prominent cellular location and abundance of the *O*‐glycan as well as the S‐layer matrix itself make them ideal candidates for influencing interbacterial or bacterium–host interactions as may occur in oral biofilms.

**Table 1:** *Tannerella forsythia* strains and their cell surface mutants cultivated in the subgingival “Zurich biofilm model”

Strains	Cell surface and glycan properties	Structure of *O*‐glycan
ATCC 43037		
Wild‐type	wild‐type; S‐layer glycan with terminal Pse residue	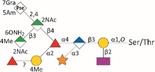
Δ*pseC (Tanf_01190)*	S‐layer glycan devoid of terminal Pse	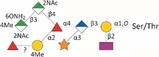
Δ*wecC (Tanf_01280)*	S‐layer glycan devoid of trisaccharide branch containing Pse and two ManNAcA residues	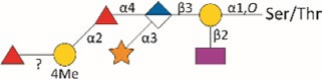
Δ*tfsAB (Tanf_03370; Tanf_03375)*	S‐layer deficient mutant; this mutant may expose R‐type lipopolysaccharide or *O*‐glycans from outer membrane glycoproteins	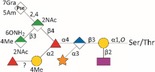
Δ*pseC*_*comp*_ *(Tanf_01190)*	reconstituted mutant Δ*pseC*	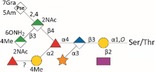
UB4		
Wild‐type	wild‐type; S‐layer glycan with terminal Leg residue	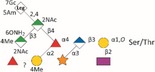
Δ*legC (TFUB4_00900)*	S‐layer glycan devoid of terminal Leg	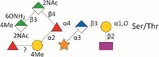
Δ*legC*_*comp*_ *(TFUB4_00900)*	reconstituted mutant Δ*legC*	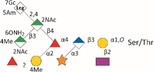

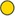
galactose; 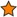
xylose; 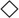
nonulosonic acid; Gra *N*‐glyceroyl; 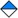 glucuronic acid; 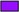 digitoxose; NAc *N*‐acetyl; Me *O‐*methyl; 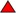
fucose; 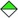
mannosaminuronic acid; Am acetamidino; Gc glycolyl

In biofilms, the physical properties of the bacterial cell surface come into play, as initial attachment by planktonic bacteria to a substrate is primarily influenced by factors such as surface charge, hydrophobicity or electrostatic interactions, whereas the formation of a stable biofilm is facilitated by specialized surface components such as flagella, fimbriae, or pili and the production of an exopolysaccharide matrix.^29^, ^30^ The oral bacterium *Streptococcus sanguis*, for instance, has been shown to largely depend on hydrophobic effect interactions for adhesion to the salivary pellicle.^31^, ^32^ *Streptococcus parasanguinis*, another early colonizer of the dental surface, requires glycosylation of the fimbria‐associated adhesin Fap1 for the formation of stable biofilms.^33^, ^34^ In *Campylobacter* spp., loss of flagellum glycosylation negatively affects the bacterium's ability to form microcolonies and, subsequently, biofilms.^35^, ^36^ In *Campylobacter jejuni,* the flagellum is heavily glycosylated by the addition of *O‐*linked pseudaminic acid (Pse) and legionaminic acid (Leg),^37^, ^38^ a feature that has been shown to orchestrate the bacterium's virulence potential.^36^

We recently found evidence that the *T. forsythia* ATCC 43037 wild‐type strain carries a modified Pse residue as a terminal constituent of the S‐layer *O‐*glycan,^26^ whereas in the clinical isolate *T. forsythia* UB4, this residue is present as its stereoisomer, Leg^39^ (Table 1, see Supplementary material, Fig. S1). Pse (5,7‐diacetamido‐3,5,7,9‐tetradeoxy‐l‐*glycero*‐l‐*manno‐*non‐2‐ulosonic acid) as well as Leg (5,7‐diacetamido‐3,5,7,9‐tetradeoxy‐d‐*glycero*‐d‐*galacto‐*non‐2‐ulosonic acid) appear to be unique to bacteria.^39^ They belong to the class of nonulosonic acids, acidic nine‐carbon (C9) α‐keto sugars, which are best represented by the sialic acid family abundantly displayed on the exterior of mammalian cells functioning in cell–cell communication and adhesion.^40^

The *T. forsythia* S‐layer has been described to facilitate adhesion to and invasion of gingival epithelial cells,^41^ suppress pro‐inflammatory cytokine production,^42^ and inhibit monospecies biofilm formation;^28^ however, without dissecting any potential contribution of the *O*‐glycan attached to the S‐layer. Honma *et al*. (43) observed an increase in *T. forsythia* biofilm formation upon deletion of a UDP‐*N*‐acetyl‐d‐mannosaminuronic dehydrogenase (WecC) – later found to cause a three‐sugar truncation of the *T. forsythia O*‐glycan^26^ (compare with Table 1) – when cells were cultivated in an untreated polystyrene culture dish.^43^ In contrast, deficiency in the *O*‐glycan's terminal nonulosonic acid in a *T. forsythia* ATCC 43037 Δ*pseC* and a *T. forsythia* UB4 Δ*legC* mutant, respectively, decreased biofilm formation on a mucin‐coated surface.^39^ Although these data together demonstrate the involvement of both S‐layer and attached sugar moieties in monospecies biofilm formation, the question arises to what extent these observations are influenced by the physical properties of the surface provided for cell attachment and, above that, demand an investigation into if and how the described effects translate into a multispecies biofilm that more adequately mirrors the *in vivo* situation. As part of a multispecies biofilm consortium, *T. forsythia* needs to interact with numerous other bacteria. How these interactions are mediated and whether they depend on the *T. forsythia* S‐layer and/or its *O*‐glycosylation has yet to be elucidated.

Based on the analysis of planktonic and monospecies biofilm growth, we employed in this study the subgingival “Zurich biofilm model”^44^ to investigate how the *T. forsythia* wild‐type strains ATCC 43037 and UB4 and defined cell surface mutants thereof perform in a multispecies consortium. Through the incorporation of 10 different species of oral bacteria in the biofilm, this *in vitro* model mimics the natural situation in the oral cavity, whereby several microbial species assemble and grow together in the form of a biofilm, and therefore poses an excellent platform to dissect the role of individual species within the community. In the *in vitro* model, the selected oral bacteria, including the three “red complex” species out of which *T. forsythia* was varied, were co‐cultivated to form biofilms on pellicle‐coated hydroxylapatite (HA) disks in saliva and serum‐containing growth medium.^12^, ^45^, ^46^, ^47^, ^48^, ^49^ *Tannerella forsythia* wild‐type strains and mutants with different cell surface glycosylation patterns as well as an S‐layer‐deficient mutant were introduced in order to monitor their biofilm growth as well as the structural behavior of the biofilm communities as a whole.

In particular, biofilms grown under these conditions were analyzed with the following aims: (i) to numerically determine cell numbers of all individual species within the bacterial consortium and the overall biofilm composition by quantitative real‐time polymerase chain reaction (qPCR) and colony‐forming unit (CFU) counts and (ii) to analyze the localization and distribution of individual species within the microbial structure through fluorescence *in situ* hybridization (FISH) using species‐specific probes against the 16S rRNA and confocal laser scanning microscopy (CLSM) analysis. This study is intended to be a first characterization of the behavior of *T. forsythia* strains with varying cell surface composition in a multispecies biofilm setting.

## 2 METHODS

### 2.1 Bacterial strains

*Tannerella forsythia* ATCC 43037 (American Type Culture Collection, Manassas, VA) and *T. forsythia* UB4 (obtained from Dr. Ashu Sharma, University of Buffalo, NY, USA) wild‐type strains and defined mutants thereof (see below) were grown anaerobically at 37°C for 4‐7 days in brain–heart infusion broth (Oxoid, Basingstoke, UK), supplemented with *N*‐acetylmuramic acid, horse serum, and 50 μg mL^−1^ gentamicin as described previously,^27^ with one passage before biofilm inoculation.

Mutants of *T. forsythia* ATCC 43037 (JUET00000000^50^) and *T. forsythia* UB4 (FMMN01000000^51^) with characterized defects in their cell surface protein glycosylation, affecting the terminal Pse (ATCC 43037) or Leg (UB4) residue, were available in our laboratory from a previous study.^39^ Briefly, *T. forsythia* ATCC 43037 ∆*pseC* (coding for a dedicated aminotransferase from the Pse biosynthesis pathway) and *T. forsythia* UB4 ∆*legC* (coding for a dedicated aminotransferase from the Leg biosynthesis pathway) mutants were constructed by chromosomal insertion of a gene knockout cassette consisting of an erythromycin resistance gene flanked by homologous upstream and downstream regions, ~1000 bp, each. The complementation cassette for *T. forsythia* mutants consisted of a chloramphenicol resistance gene flanked by a homologous ~1000‐bp upstream region, the gene of interest and a ~1000‐bp downstream region. The *T. forsythia* ATCC 43037 *∆wecC* mutant, which lacks a trisaccharide glycan branch including the Pse residue, was obtained from Dr. Ashu Sharma. In addition to that, the S‐layer‐deficient mutant *T. forsythia* ATCC 43037 *∆tfsAB* 41 was included in this study. This mutant lacks S‐layer glycans due to the absence of the S‐layer, but may expose underlying R‐type lipopolysaccharide^52^ or even *O*‐glycans present on outer membrane glycoproteins that become exposed upon removal of the S‐layer.^53^ All *T. forsythia* strains and mutants used in this study, together with their cell surface composition, are summarized in Table 1.

### 2.2 Monospecies biofilm growth of *T. forsythia*

The monospecies biofilm behavior of all *T. forsythia* strains and mutants included in this study was analyzed in a microtiter plate assay.^39^ In brief, bacteria were passaged once before biofilm inoculation at an optical density at 600 nm (OD_600_) of 0.05 and grown anaerobically for 6 days in 1 mL of half‐concentrated brain–heart infusion medium, with supplements as above,^27^ in 24‐well polystyrene plates (STARLAB) coated with 5 mg mL^−1^ mucin (from bovine submaxillary gland; Sigma‐Aldrich, Vienna, Austria) solution (in 0.1 mol L^−1^ sodium acetate buffer pH 4.5). In each experiment, two wells were used to determine the total cells of each strain and mutant, sterile medium served as negative control. For biofilm quantification, medium and planktonic cells were removed and the wells were washed once with 500 μL of PBS. Subsequently, biofilms were resuspended in 1 mL of PBS and the OD_600_ of the biofilm cell suspension was measured. Biofilm values were normalized to the corresponding absorbance (OD_600_) of the total cells. Data represent mean values ±SD of four independent experiments with three replicates each and were analyzed by the unpaired Student's *t*‐test.

### 2.3 Multispecies biofilm cultivation

To set‐up the “Zurich subgingival biofilm model”, *T. forsythia* wild‐type strains and defined cell surface mutants thereof (Table 1) were co‐cultivated with the following organisms: *Prevotella intermedia* ATCC 25611^T^ (OMZ278), *Campylobacter rectus* (OMZ388), *Veillonella dispar* ATCC 17748^T^ (OMZ493), *Fusobacterium nucleatum* (OMZ598), *Streptococcus oralis* SK248 (OMZ607), *Streptococcus anginosus* ATCC 9895 (OMZ871), *Actinomyces oris* (OMZ745), *Porphyromonas gingivalis* (OMZ925), and *Treponema denticola* ATCC 35405 (OMZ661). Each biofilm contained nine standard subgingival species plus one of the eight *T. forsythia* strains and mutants. Biofilm bacteria were maintained as described previously.^44^

For biofilm formation, bacterial cultures at an OD_600_ of 1.0 were mixed at equal volumes and 200 μL of this cell suspension was used to inoculate 1.6 mL of growth medium (60% pooled saliva, 10% fetal bovine serum [Sigma], 30% modified fluid universal medium)^54^ for biofilm formation on sintered pellicle‐coated HA disks (9 mm in diameter; Clarkson Chromatography Products, South Williamsport, PA) positioned in 24‐well polystyrene tissue‐culture plates. The medium was changed after 16 and 24 hours and disks were dip‐washed in 0.9% NaCl three times a day. After incubating anaerobically at 37°C for 64 hours, biofilms were dip‐washed once more and either harvested by vigorous vortexing for 2 minutes in 0.9% NaCl or fixed for 1 hour at 4°C in 4% paraformaldehyde solution (Merck, Darmstadt, Germany) for FISH.

### 2.4 Quantitative analysis

Cell numbers were determined by serial dilution plating and CFU counting as well as qPCR on genomic DNA purified from biofilm samples. Cell numbers were taken as a measure for the bacterial growth rate within the biofilm.

For CFU counts, biofilm suspensions were diluted 1:10^4^ and 1:10^5^ in 0.9% NaCl and plated on selective agar plates (Table 2) using a spiral diluter. For the more fastidious strains – i.e. *T. denticola*,* C. rectus*, and *T. forsythia* – cell numbers were determined by qPCR only.

**Table 2:** Selective agar plates used for colony‐forming unit counting

Selective agar plates	Organism
Mitis Salivarius Agar (Difco)+1% sodium tellurite solution	*Streptococcus anginosus*,* Streptococcus oralis*
Columbia Blood Agar (Oxoid)+5% horse blood (Sigma)	*Actinomyces oris, Veillonella dispar* total CFU
Fastidious Anaerobe Agar (BAG)+1 mg L^−1^ erythromycin (Sigma), 4 mg L^−1^ vancomycin (Sigma), 1 mg L^−1^ norfloxacin (Sigma)	*Fusobacterium nucleatum*
Columbia Blood Agar+5% horse blood (Sigma), 80 mg L^−1^ phosphomycin (Sigma)	*Prevotella intermedia, Porphyromonas gingivalis*

For qPCR, bacterial genomic DNA was extracted from 500 μl of biofilm suspension using the GenEluteTM Bacterial Genomic DNA Kit (Sigma) and qPCR was performed on an ABI Prism SDS 7000 device (Applied Biosystems, Foster City, CA) according to Ammann *et al*.^47^ Each sample was analyzed using species‐specific primers amplifying the 16S rRNA gene.^47^ For each species, a standard curve was generated and the sample DNA concentration was calculated from the obtained quantification cycle (Cq) values. The abundance of each organism in the biofilm was calculated using the respective theoretical genome weight.^47^ Cell numbers per biofilm were determined in three independent experiments with three technical replicates for each biofilm. Statistical significance was tested by analysis of variance (Tukey's post‐hoc test for multiple comparisons, *P*≤.05) using graphpad prism version 7.00 for Windows (GraphPad Software, La Jolla, CA).

### 2.5 Structural analysis of biofilms

FISH staining was performed according to the protocol established by Thurnheer *et al*.^55^ using the probe combinations listed in Table 3. In brief, after fixation, biofilm samples were pre‐hybridized in hybridization buffer (0.9 mol L^−1^ NaCl, 20 mmol L^−1^ Tris–HCl, [pH 7.5], 0.01% sodium dodecyl sulfate, formamide (35%‐40%) at 46°C, for 15 minutes, followed by 3 hours of hybridization with specific oligonucleotide probes.^45^ Samples were washed in wash buffer (20 mmol L^−1^ Tris–HCl [pH 7.5], 5 mmol L^−1^ ethylene diaminetetraacetic acid, 0.01% sodium dodecyl sulfate, 46‐70 mmol L^−1^ NaCl) for 45 minutes at 48°C. For CLSM and image analysis, the samples were counterstained with a mixture of 3 μmol L^−1^ YoPro‐1 iodide (Invitrogen, Carlsbad, CA) and 15 μmol L^−1^ Sytox Green (Invitrogen) and embedded in Mowiol^56^ for confocal microscopy.

**Table 3:** Combinations of 16S rRNA probes used for fluorescence *in situ* hybridization staining of individual bacterial species

Probes	Target species	FA (%)^a^	NaCl (mmol L^−1^)^b^	Reference
*Tfor‐997‐Cy3/Pging1006‐2‐prop‐Cy5*	*Tannerella forsythia/Porphyromonas gingivalis*	40	46	44, 75
*Tfor‐997‐Cy3/Pging1006 ‐Cy5*	*T. forsythia/P. gingivalis*	40	46	44, 75
*Tfor‐997‐Cy3/TrepG1‐679‐Cy5*	*T. forsythia/Treponema denticola*	40	46	75, 76
*Tfor‐997‐Cy3/FUS‐664‐Cy5*	*T. forsythia/Fusobacterium nucleatum*	40	46	55, 75
*Tfor‐997‐Cy3/CAMP655‐Cy5*	*T. forsythia/Campylobacter rectus*	35	70	44, 75
*Tfor‐997‐Cy3/Pging1006‐2‐prop‐Cy5*	*T. forsythia/P. gingivalis*	40	46	44, 75
*Tfor‐997‐Cy3/Pging1006 ‐Cy5*	*T. forsythia/P. gingivalis*	40	46	44, 75
*Tfor‐997‐Cy3/TrepG1‐679‐Cy5*	*T. forsythia/T. denticola*	40	46	75, 76
*Tfor‐997‐Cy3/FUS‐664‐Cy5*	*T. forsythia/F. nucleatum*	40	46	55, 75
*Tfor‐997‐Cy3/CAMP655‐Cy5*	*T.forsythia/C. rectus*	35	70	44, 75

a Formamide concentration used in the hybridization buffer.

b NaCl concentration in the wash buffer.

The architecture of the biofilms was analyzed using CLSM. For each of the eight *T. forsythia* strains and mutants, a minimum of three disks carrying fluorescently labeled biofilms was analyzed using a Leica SP‐5 microscope (Center of Microscopy and Image Analysis of the University of Zürich). Images were captured using a 100× objective and processed with imaris 7.4.0 Software (Bitplane, Zürich, Switzerland). Presented CSLM images (Figures 3 5 6) are snapshots of the biofilm structures present on the HA disks and the depicted structures represent a comprehensive collection of *T. forsythia* biofilm behavior observed during sampling.

## 3 RESULTS

### 3.1 Monospecies biofilm formation of *T. forsythia* wild‐type strains and mutants

**Figure 1:**
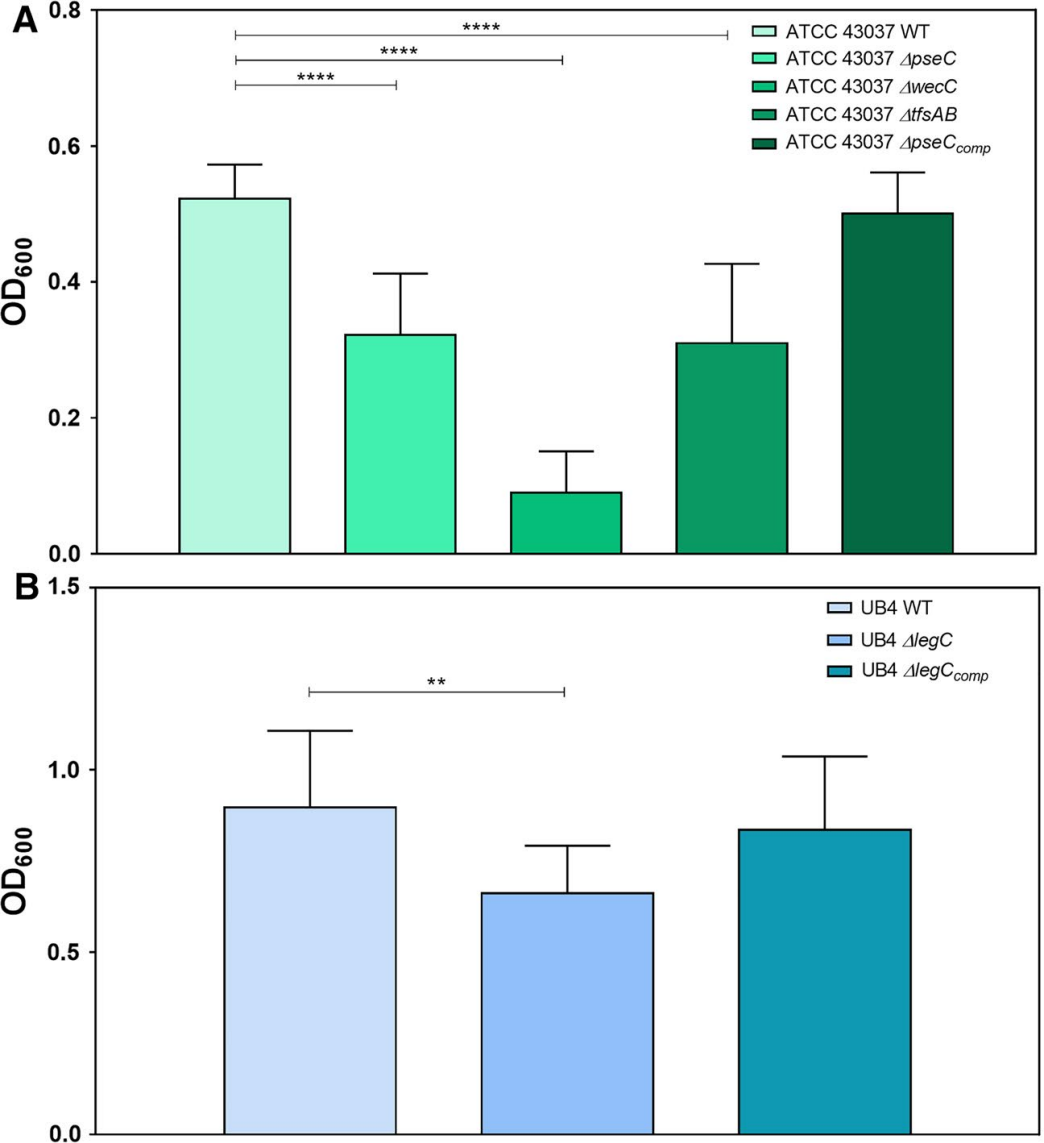
Monospecies biofilm formation of *Tannerella forsythia* wild‐type and mutant strains. (A) Biofilm formation of *T. forsythia* ATCC 43037 wild‐type compared with its mutants ATCC 43037 Δ*pseC*, Δ*wecC*, Δ*tfsAB* and the complemented mutant Δ*pseC*_comp_. (B) Biofilm formation of *T. forsythia* UB4 wild‐type compared with its mutant UB4 Δ*legC* and the complemented mutant Δ*legC*_comp_. Mean values ±SD of four independent experiments with three replicates, each, are shown. Asterisks (**) indicate significant differences between samples as determined by the unpaired Student's *t*‐test (*P*≤.01)

Based on the observations that deficiency in the protein *O*‐glycan's terminal nonulosonic acid triggers a decrease in biofilm formation of *T. forsythia* ATCC 43037 *∆pseC* and *T. forsythia* UB4 *∆legC* on a mucin‐coated surface^39^ and that *T. forsythia* ATCC 43037 *∆wecC* possessing an even more truncated *O*‐glycan forms more biofilm on untreated plates,^43^ the biofilm formation capacity of all these strains was compared here in one microtiter plate assay, where the plates were coated with mucin to mimic the native situation on the tooth surface, and biofilm growth was quantified by OD_600_ measurement of biofilm cells and normalized to the corresponding total cell mass for each strain. In our setting, any manipulation of the cell surface decreased the capacity of the bacteria to form biofilms, as was evident in the absence of the S‐layer (*T. forsythia* ATCC 43037 *∆tfsAB*), of the Pse‐(ManNAcA)_2_ *O*‐glycan branch (*T. forsythia* ATCC 43037 *∆wecC*) as well as of the terminal nonulosonic acid alone, i.e. Pse in *T. forsythia* ATCC 43037 *∆pseC* and Leg in *T. forsythia* UB4 *∆legC* (Figure 1). More precisely, biofilms of the ATCC 43037 strain reached an average maximum OD_600_ of 0.52 ± 0.05 after 6 days of cultivation, whereas biofilm growth of the *∆pseC* and *∆tfsAB* mutants was reduced by 1.6‐fold, and in the case of the *∆wecC* mutant even by five‐fold*. Tannerella forsythia* UB4 wild‐type biofilms reached an average maximum OD_600_ of 0.89 ± 0.21 and also here, the biofilm growth was reduced 1.3‐fold in the nonulosonic acid‐deficient mutant *∆legC*. In both nonulosonic acid mutants, the growth behavior in the biofilm was restored to the levels of the respective parent strain, with an average maximum OD_600_ of 0.50 ± 0.06 for *∆pseC*_comp_ and 0.84 ± 0.20 for *∆legC*_comp_ (Figure 1).

All deletion mutants also showed slower planktonic growth in liquid culture, as concluded from the determination of growth curves and doubling times (see Supplementary material, Fig. S2 and Table S1). These obvious growth defects might result from pleiotropic effects due to the genetic manipulation of the strains rather than from changes of the bacterial cell surface, even though during planktonic growth, both complemented strains performed in a similar way to the parent strain in terms of doubling times, with *∆pseC*_comp_ vs ATCC 43037 wild‐type revealing doubling times of 14.99 ± 0.83 hours and 14.41 ± 0.54 hours, and *∆legC*_comp_ vs UB4 revealing a slight increase in doubling time (12.28 ± 0.25 hours vs 16.75 ± 3.97 hours) (see Supplementary material, Fig. S2 and Table S1).

### 3.2 Determination of total biofilm cell numbers in the presence of *T. forsythia* strains and mutants in the subgingival “Zurich biofilm model”

Total cell numbers in biofilms including nine bacterial species routinely used in the subgingival “Zurich biofilm model” plus one *T. forsythia* wild‐type strain (*T. forsythia* ATCC 43037 or UB4) or mutant lacking certain sugar residues (*T. forsythia* ATCC 43037 *∆pseC*,* T. forsythia* ATCC 43037 *∆wecC*,* T. forsythia* UB4 *∆legC*) or the whole S‐layer (*T. forsythia* ATCC 43037 *∆tfsAB*) were analyzed by quantifying the cell numbers of each of the 10 species.

**Figure 2:**
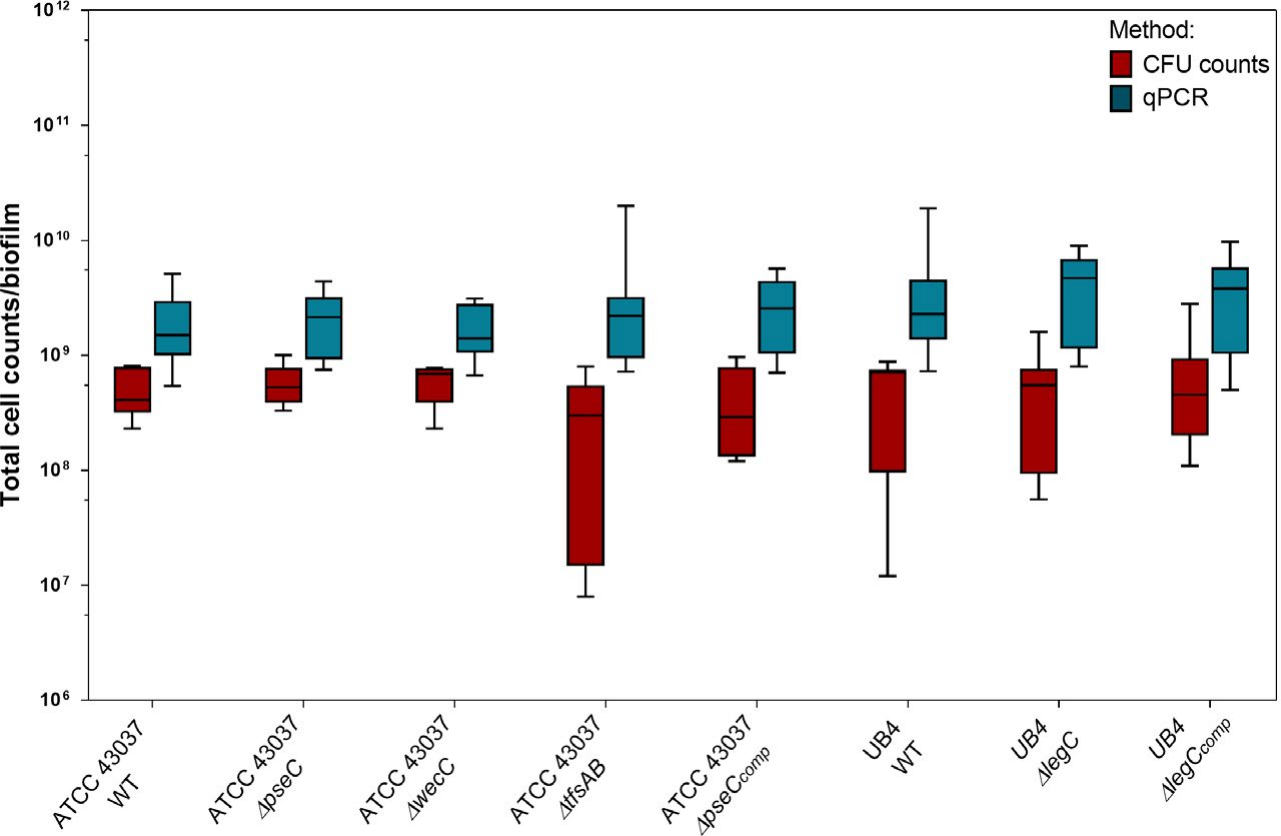
Comparison of colony‐forming unit (CFU) counting and quantitative polymerase chain reaction (qPCR) for *Tannerella forsythia* wild‐type strains and mutants in the subgingival “Zurich biofilm”. Total bacteria for 10‐species biofilms with different *T. forsythia* strains and mutants enumerated by CFU counts (red boxes) and qPCR (blue boxes) for three independent experiments with three technical replicates, each, are shown (Whiskers boxplots 5th to 95th centile)

The total cell number per biofilm was not significantly affected, regardless of which *T. forsythia* strain or mutant had been incorporated into the biofilm (Figure 2). When comparing the total cell number of all biofilm bacteria as determined by strain‐specific qPCR and CFU counts, the latter resulted in lower cell numbers, as only viable cells were enumerable. Both methods, however, provided reproducible results for each of the nine disks that were analyzed for each of the eight *T. forsythia* strains and mutants included in this study.

### 3.3 Influence of *T. forsythia* wild‐type strains on composition and structure of the subgingival “Zurich biofilm model”

First, the multispecies biofilm behavior of the *T. forsythia* wild‐type strains ATCC 43037 and UB4 was compared with regard to bacterial growth and localization in the 10‐species biofilm.

**Figure 3:**
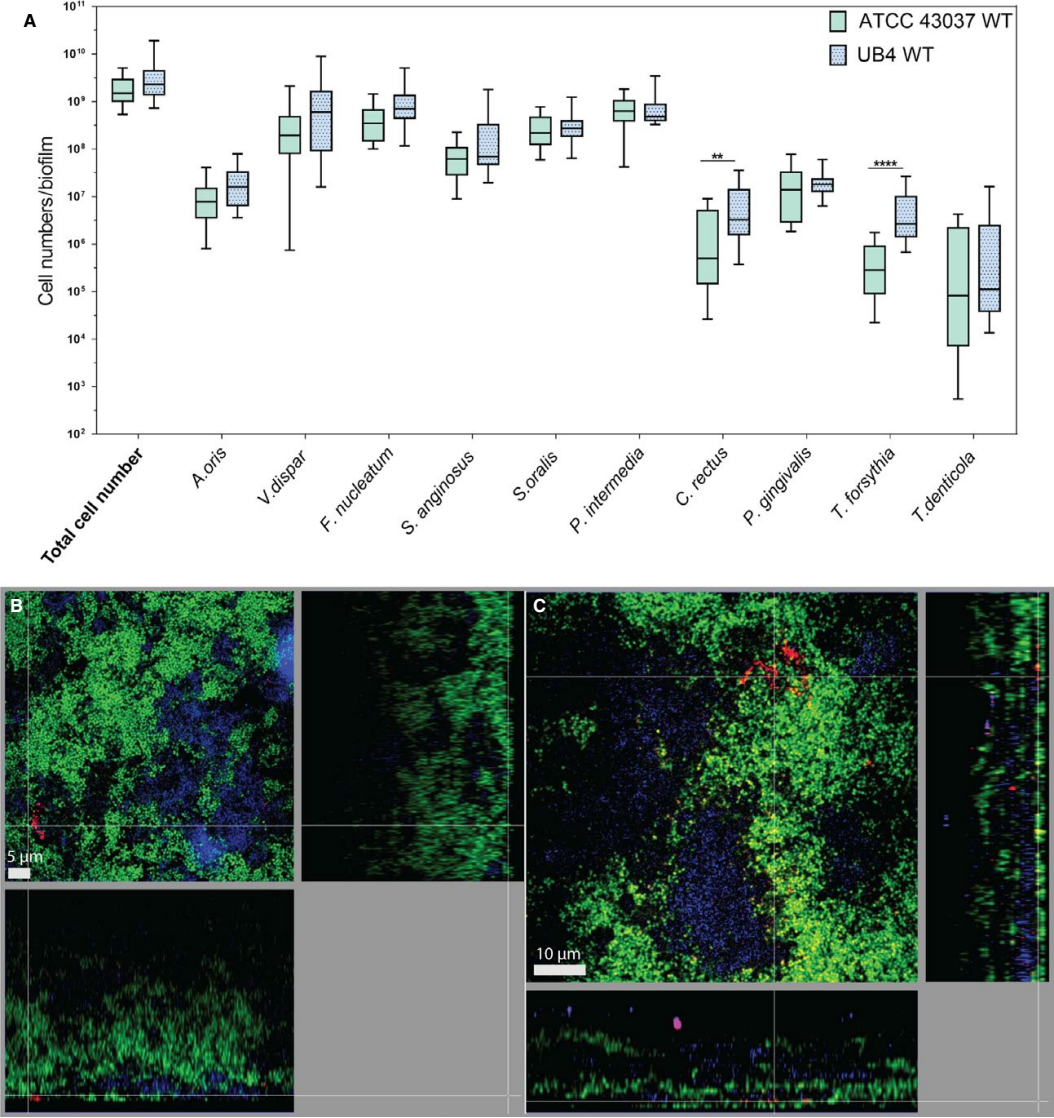
Comparison of 10‐species biofilms with two *Tannerella forsythia* wild‐type strains. (A) Whiskers boxplots (5th to 95th centile) show bacterial numbers determined by quantitative real‐time PCR from three independent experiments. Asterisk (*) indicates a statistically significant difference (*P*≤.05) between groups. The two groups represent biofilms with either *T. forsythia* ATCC 43037 wild‐type or *T. forsythia* UB4 wild‐type. (B, C) Fluorescence *in situ* hybridization stainings of fixed biofilms showing the localization of ATCC 43037 wild‐type (B) and UB4 wild‐type (C). Red/yellow: *T. forsythia;* cyan: *Porphyromonas gingivalis*, green: non‐hybridized cells (DNA staining YoPro‐1+Sytox). Here a representative area for one disk each is shown with a top view in the left panel and a side view with the biofilm–disk interface directed towards the top view; scale bars 5 μm (B) and 10 μm (C)

For quantitative analysis, the cell number of each individual species in the biofilm was determined by qPCR after 64 hours of incubation. The total cell number of all species, except for *C. rectus* OMZ388 (see below), was not affected by the incorporation of the different *T. forsythia* strains, but there was a clear difference in the biofilm growth of the *T. forsythia* strains (Figure 3A). In accordance with the results observed in monospecies biofilms (Figure 1), also in the 10‐species consortium, *T. forsythia* UB4 seemed to perform better with mean cell numbers higher by 11.9‐fold when compared with the mean cell numbers of *T. forsythia* ATCC 43037 (Figure 3A), as determined by qPCR. This coincided with *C. rectus* OMZ388 to be found at significantly higher levels (3.6‐fold) in biofilms containing strain UB4 as determined by analysis of variance (*P*≤.01).

The influence of the two *T. forsythia* wild‐type strains on the biofilm structure and their localization were determined by CLSM. *Tannerella forsythia* ATCC 43037 tended to be primarily localized at the outer biofilm surface in the form of clearly visible cell clusters (Figure 3B). In contrast, *T. forsythia* UB4 was found in the form of microcolonies as well as singly dispersed close to the biofilm surface and in small clusters in deeper layers of the biofilm (Figure 3C).

### 3.4 Analysis of *T. forsythia* cell surface mutants in the subgingival biofilm

#### 3.4.1 Quantitative analysis

In order to assess to what extent the difference in biofilm growth of *T. forsythia* strains ATCC 43037 and UB4 (see above) was influenced by their cell surface composition, defined mutants of either strain (Table 1) were incorporated into the biofilm and their cell numbers were again determined via qPCR.

**Figure 4:**
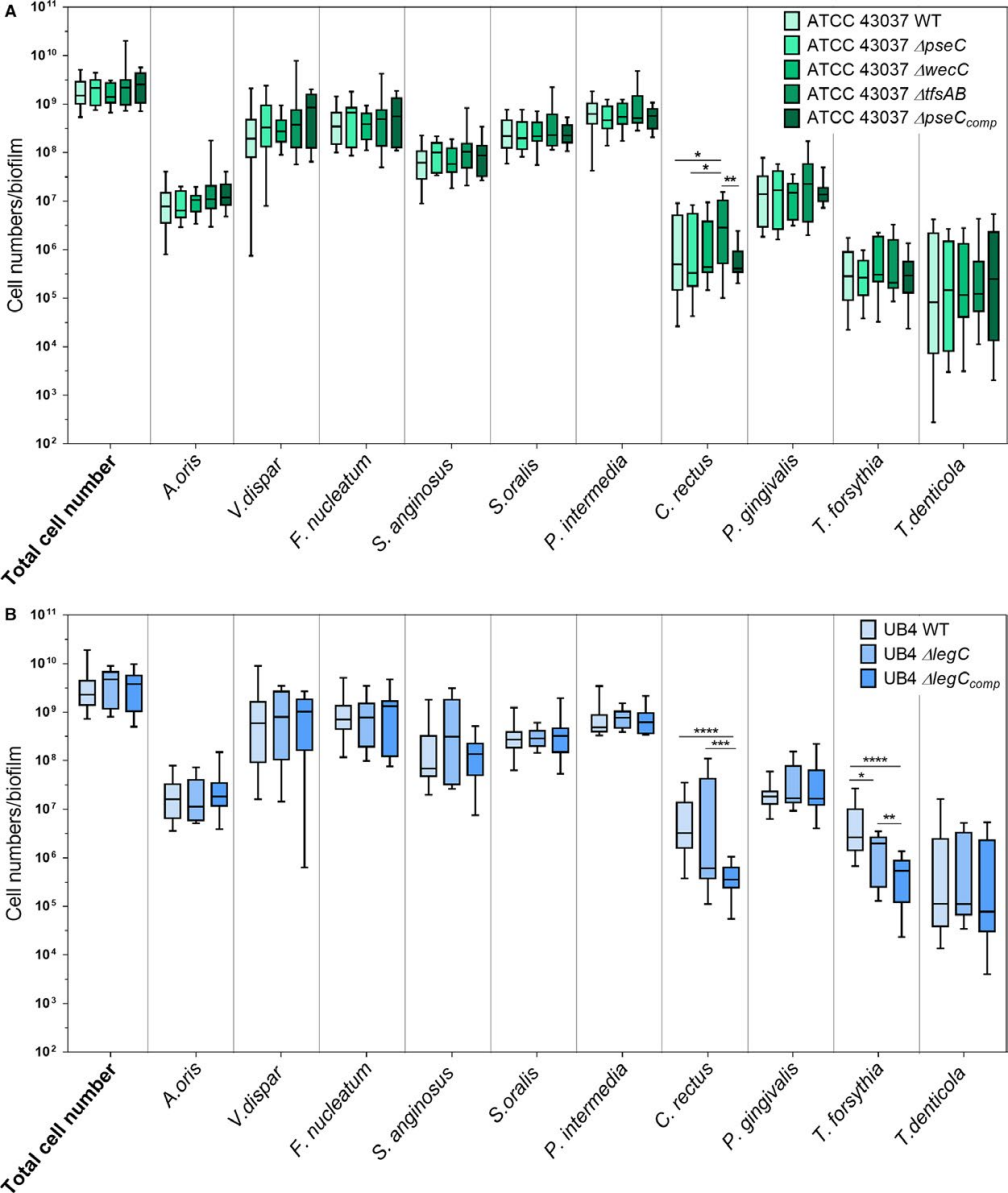
Box plots showing cell numbers of all species determined by quantitative real‐time PCR for biofilms with *Tannerella forsythia* ATCC 43037 wild‐type or mutants *(∆pseC*,* ∆wecC*,* ∆tfsAB*,* ∆pseC*_comp_) (A) and UB4 wild‐type or mutants *(∆legC*,* ∆legC*_comp_), respectively (B). Data derived from three independent experiments were plotted on a logarithmic scale. Asterisk (*) indicates significant differences (*P*≤.05) between the groups

Contrary to their performance in monospecies biofilms (Figure 1) and their slower planktonic growth (see Supplementary material, Fig. S2) in the 10‐species consortium, the *T. forsythia* ATCC 43037 mutants behaved in a very similar way to the parent strain, and neither the lack of the terminal Pse residue (*∆pseC*) nor the lack of the trisaccharide branch (*∆wecC*) of the S‐layer *O*‐glycan significantly affected the growth of *T. forsythia* or the other species of the biofilm (Figure 4A). The same was observed for the reconstituted strain ATCC 43037 *∆pseC*_comp_. Interestingly, the absence of the S‐layer in the *∆tfsAB* mutant, although not affecting growth of *T. forsythia* itself, led to a strong increase in the growth of *C. rectus* OMZ388 in the biofilm (Figure 4A) when compared with biofilms with *T. forsythia* ATCC 43037 wild‐type or *∆pseC*, indicating that the loss of the S‐layer causes a growth benefit for *C. rectus* OMZ388 in these biofilms. As a control, the complemented mutant *T. forsythia* ATCC 43037 *∆pseC*_comp_ reverted *C. rectus* OMZ388 cell numbers back to the wild‐type level (with a non‐significant reduction of *C. rectus* cell numbers).

In contrast to the almost identical performance of *T. forsythia* ATCC 43037 wild‐type and mutants in the multispecies biofilm, genetic manipulation of *T. forsythia* UB4 *(*i.e. *T. forsythia* UB4 *∆legC* and *∆legC*_comp_) resulted in a decrease in cell numbers in multispecies biofilms. The cell number of *T. forsythia* UB4 *∆legC* was significantly decreased in the biofilm when compared with the parent strain, a fact that had already been observed in monospecies biofilms (Figure 1). Contrary to its behavior in monospecies biofilms, in the multispecies community, the reconstituted strain *∆legC*_comp_ could not restore the parent phenotype (Figure 4B).

As described before, at the high levels of *T. forsythia* UB4 wild‐type that developed in the biofilm, cell numbers of *C. rectus* OMZ388 were elevated in comparison with biofilms harboring *T. forsythia* ATCC 43037. In biofilms containing the *∆legC* mutant this effect was less pronounced, with *C. rectus* OMZ388 mean cell numbers being significantly decreased by 1.4‐fold when compared with biofilms with UB4 wild‐type (*P*≤.001) (Figure 4B). In the presence of the complemented strain *∆legC*_comp_, the growth of *C. rectus* OMZ388 was significantly reduced when compared with biofilms harboring UB4 wild‐type or *∆legC* (*P*≤.001) (Figure 4B). Given that in monospecies biofilm experiments as well as during planktonic growth, UB4 *∆legC*_comp_ was shown to behave in the same way as its parent strain (Figure 1, see Supplementary material, Fig. S2), its impaired growth in the multispecies community suggests that the modification of this gene locus has a pleiotropic effect causing a growth defect in the environment of the multispecies biofilm.

#### 3.4.2 Evaluation of the biofilm structure by CLSM

Since changes in the cell surface composition of *T. forsythia* did not affect the numeric composition of the 10‐species biofilms, FISH staining and CLSM analysis were performed for a qualitative evaluation of the biofilm structure.

**Figure 5:**
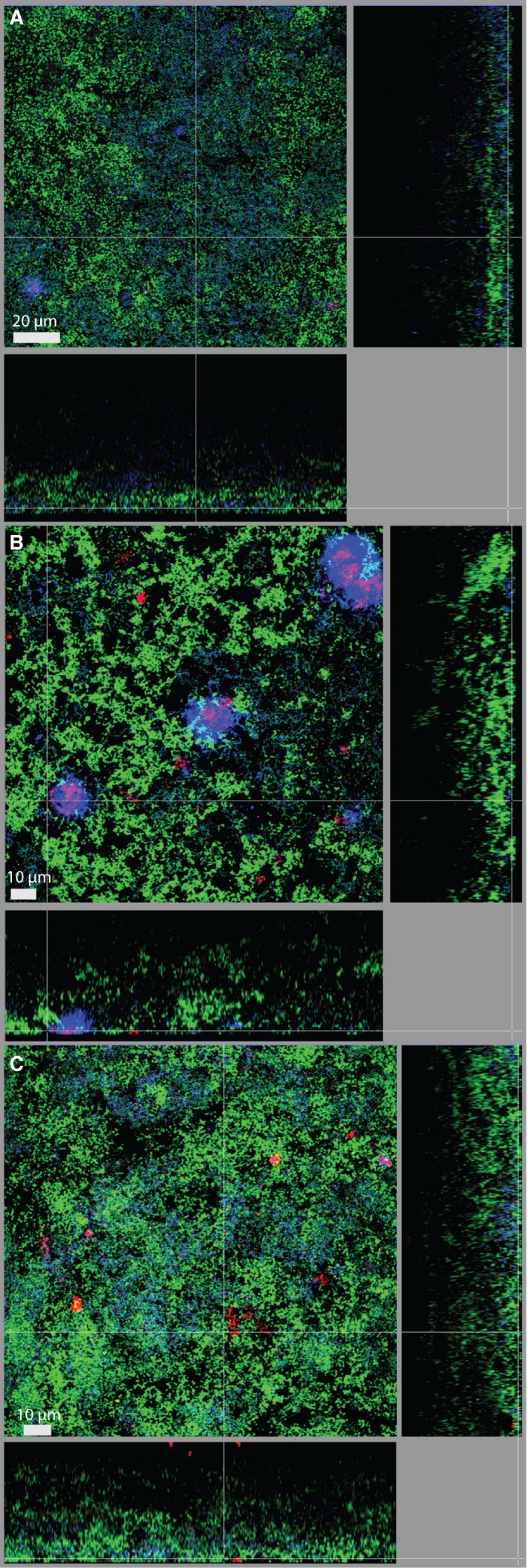
Fluorescence *in situ* hybridization staining of biofilms harboring *Tannerella forsythia* ATCC 43037 mutants (A) *∆pseC*, (B) *∆wecC*, and (C) *∆tfsAB*. Red: *T. forsythia*, cyan: *Porphyromonas gingivalis*, green: non‐hybridized cells (DNA staining YoPro‐1+Sytox). Scale bars 20 μm (A) and 10 μm (B, C)

Similar to the *T. forsythia* ATCC 43037 wild‐type, both the ATCC 43037 *∆pseC* and the ATCC 43037 *∆wecC* mutants were detected at the biofilm surface. Whereas *∆pseC* was also found singly dispersed and in pronounced cell clusters at the biofilm surface, with only very few cells being detected (Figure 5A), *∆wecC* formed dense superficial clusters (Figure 5B). The S‐layer‐deficient mutant *T. forsythia* ATCC 43037 *∆tfsAB* was observed as small microcolonies scattered along the surface as well as in the form of single cells dispersed in the upper layers of the biofilm (Figure 5C).

*Tannerella forsythia* ATCC 43037 *∆wecC* formed clearly distinguishable aggregates with *P. gingivalis* OMZ925, an effect that was not observed for the other *T. forsythia* strains and mutants analyzed. In contrast to biofilms incorporating other *T. forsythia* strains and mutants*,* in *∆wecC* biofilms, cells appeared to grow less dense, as seen by YoPro‐1+Sytox staining of non‐hybridized bacteria. *Porphyromonas gingivalis* OMZ925 appeared to have changed its localization, being detected predominantly at the biofilm surface. This potentially direct interaction of *P. gingivalis* OMZ925 with the *∆wecC* mutant was followed up in co‐aggregation studies (see Supplementary material, Fig. S3B). *Porphyromonas gingivalis* OMZ925 coaggregated with all *T. forsythia* strains at different levels. Significant differences between wild‐type and mutant strains or a distinct affinity of *P. gingivalis* OMZ925 for the *∆wecC* mutant could not be observed in these assays. The truncation of the *O*‐glycan, however, was found to affect the autoaggregation of *T. forsythia* (see Supplementary material, Fig. S3A). This could be observed as a strong decrease of the OD_600_ of the cell suspensions and higher percentage of autoaggregation of ATCC 43037 *∆pseC* (38.1%) compared with *T. forsythia* ATCC 43037 wild‐type (1.5%) but also when compared with *∆wecC* (18.1%), *∆tfsAB* (4.8%) and *∆pseC*_comp_ (4.7%).

**Figure 6:**
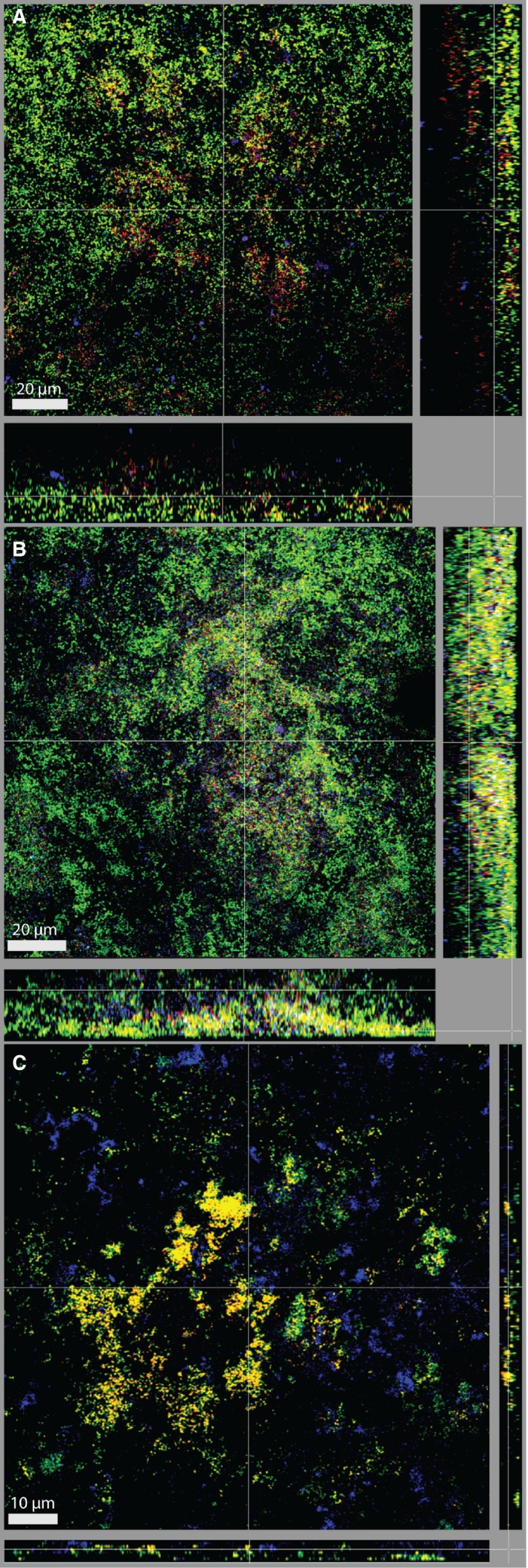
Dual fluorescence *in situ* hybridization staining of *Tannerella forsythia* and *Campylobacter rectus* for biofilms harboring ATCC 43037 wild‐type (A), UB4 wild‐type (B), and ATCC 43037 *∆tfsAB* (C). Red/yellow: *T. forsythia*, cyan: *C. rectus;* green: non‐hybridized cells (DNA staining YoPro‐1+Sytox). Scale bars 20 μm (A, B) and 15 μm (C)

Given the demonstrated growth‐promoting effect of *T. forsythia* UB4 wild‐type (Figure 3A) and *T. forsythia* ATCC 43037 *∆tfsAB* (Figure 3A) on *C. rectus* OMZ388, dual FISH stainings were performed to determine a possible coaggregation of these species (Figure 6). In the section of the biofilm shown in the CLSM images, a high number of *T. forsythia* cells was detected for both ATCC 43037 and UB4 wild‐type strains. Both were present as single cells throughout the whole biofilm structure as well as in small clusters close to the biofilm surface in the case of the ATCC 43037 wild‐type (Figure 6A) and in deeper layers for the UB4 wild‐type (Figure 6B), as had been found before (Figure 3B, C). The S‐layer mutant *∆tfsAB* was present in the form of clusters in close proximity to the HA‐disc surface in a relatively thin section of the biofilm (Figure 6B). *Campylobacter rectus* OMZ388 cells appeared in the form of irregularly interspersed microcolonies in all layers of the biofilm.

Although it was obvious that, compared with the ATCC 43037 wild‐type strain (Figure 6A), *C. rectus* OMZ388 cell numbers in the biofilm were elevated in the presence of the UB4 wild‐type strain (Figure 6B) and the ATCC 43037 S‐layer mutant *∆tfsAB* (Figure 6C), co‐localization, which would be a prerequisite of a direct interaction between the bacteria, could not be observed. Also, co‐aggregation assays did not show a direct interaction between *C. rectus* OMZ388 and the ATCC 43037 S‐layer mutant or UB4 wild‐type strain (see Supplementary material, Fig. S3C). Here, as for *P. gingivalis* OMZ925, aggregation was elevated only with the nonulosonic‐acid‐deficient strains *T. forsythia* ATCC 43037 *∆pseC* (14.3%) and *T. forsythia* UB4 *∆legC* (9.5%) when compared with ATCC 43037 wild‐type (4.8%) and UB4 wild‐type (4.1%) (see Supplementary material, Fig. S3C).

## 4 DISCUSSION

The purpose of this study was to analyze different *T. forsythia* wild‐type strains and selected mutants thereof with defined differences in cell surface composition with regard to their behavior in a multispecies biofilm community. First we showed that the monospecies biofilm lifestyle of *T. forsythia* was clearly influenced by its S‐layer and attached *O*‐glycan. Alteration of the *T. forsythia* cell surface composition significantly reduced the capability of the bacterium to form monospecies biofilms as evidenced previously with the nonulosonic‐acid‐deficient mutants ATCC 43037 *∆pseC* and *T. forsythia* UB4 *∆legC*.^39^ In this study, this effect was confirmed for a mutant with an even more truncated *O*‐glycan ATCC 43037 *∆wecC* as well as for the S‐layer‐deficient mutant ATCC43037 *∆tfsAB* (Figure 1). For the nonulosonic‐acid‐deficient mutants, biofilm formation could be fully restored in the complemented mutants, suggesting a direct correlation between loss of the terminal sugar residue and reduced biofilm formation. These data were derived from biofilm experiments using mucin‐coated polystyrene plates and are contradictory to previous findings by others, where on untreated polystyrene, biofilm formation was enhanced for the ATCC 43037 *∆wecC* mutant.^57^ In their natural habitat, mucin provides an initial adhesion site and nutrient source for bacteria and fosters biofilm growth.^58^ Mucin coating introduces highly hydrophilic properties to the otherwise hydrophobic polystyrene surface.^59^ Bacterial adhesion and interaction is influenced by hydrophobic interactions as well as steric forces and charge effects.^60^ The decrease of biofilm formation of strains that lack one (*∆pseC, ∆legC*) or more (*∆*wecC) charged sugar residues on a hydrophilic surface documented in this study vs the previously observed opposite effect on a hydrophobic surface^57^ shows that biofilm behavior is decisively influenced by the properties of the surface provided for attachment.

In this study, we investigated polymicrobial biofilms that approximate the native situation in the oral cavity much better than a planktonic or monospecies biofilm culture and, therefore, constitute an ideal system to examine the growth performance of individual species and strains. When introduced into *in vitro* 10‐species subgingival biofilms, alteration of the bacterial cell surface composition as present in the defined mutants did not impair the growth behavior of *T. forsythia* in terms of cell numbers per biofilm (Figure 4A). Interestingly, at the wild‐type level, *T. forsythia* UB4 occurred in higher numbers than *T. forsythia* ATCC 43037 (Figures 1, 3A, 4A), which may indicate a better adaptation of *T. forsythia* UB4 to the niche.

Comparison of the planktonic growth of both *T. forsythia* ATCC 43037 and UB4 wild‐type strains supported these observations, as UB4 was found to have a shorter generation time and grew to a higher OD_600_ before reaching the stationary phase in comparison with strain ATCC 43037 (see Supplementary material, Fig. S2). To our knowledge the data presented here constitute a first description of the different growth characteristics of these two *T. forsythia* strains in biofilm settings as well as in planktonic form. A preliminary bioinformatic analysis of the genomes of different *T. forsythia* isolates available in databases reflects the variability in the genetic make‐up for either Leg or Pse biosynthesis.^39^ Considering the differences in biofilm behavior of *T. forsythia* ATCC 43037 and UB4 wild‐type and that Leg is a better mimic of the biologically important sialic acid than Pse, this might suggest that the presence of either nonulosonic acid could reflect the adaptation of *T. forsythia* strains to different oral microenvironments.

Whereas in the multispecies biofilms the overall cell numbers remained relatively constant, the distribution of *T. forsythia* changed depending on the bacterium's cell surface composition. Neither the S‐layer nor its glycosylation seemed to be required for the bacterium to establish itself in the multispecies community. However, changes thereof influenced *T. forsythia*'s autoaggregation, which was enhanced upon truncation of the *O*‐glycan in the mutants *T. forsythia* ATCC 43037 *∆pseC*,* T. forsythia* ATCC 43037 *∆wecC*, and *T. forsythia* UB4 *∆legC* (see Supplementary material, Fig. S3). Alteration of the cell surface might, therefore, change the way that cells interact with each other within the microcolonies and multispecies cell aggregations. As cell surface glycosylation affected biofilm formation on a mucin‐coated surface (Figure 1), it is tempting to speculate that the decreased ability to adhere to the heavily sialylated salivary glycoprotein mucin in a monospecies biofilm setting is mirrored in the multispecies consortium in a way that *T. forsythia* cell surface mutants might exhibit an altered capability to adhere to sialic‐acid‐like structures present on other oral bacteria, such as streptococci or *Campylobacter* species^37^, ^38^ and, thereby, vary their localization within the multispecies consortium.

From the other bacterial species in the multispecies biofilm, *C. rectus* OMZ388 seems to be strongly affected by the *T. forsythia* cell surface composition. Upon presence of the *T. forsythia* ATCC 43073 *∆tfsAB* mutant, which is deficient for the S‐layer and, hence, also the attached *O*‐glycans, *C. rectus* OMZ388 was increased in its cell numbers per biofilm (Figures 3A, 4A, 6C). Hence, it is conceivable that in the native multispecies situation, the glycosylated S‐layer as an entity (ATCC 43037) might have a regulatory role in keeping *C. rectus* cell numbers below a certain threshold. In fact, a previous proteomic analysis of *T. forsythia* biofilms identified the two S‐layer proteins TfsA and TfsB to be upregulated in comparison with the planktonic cells,^61^ which underlines the importance of the S‐layer for the biofilm lifestyle of the bacterium. The causative factors and underlying mechanism for the increased growth of *C. rectus* OMZ388 in biofilms harboring the *T. forsythia* ATCC 43037 Δ*tfsAB* mutant still await further investigation. Structural analysis of these biofilms and coaggregation assays performed so far suggest that the observed growth effect is independent of a direct interaction between the two species (Figure 6, and see Supplementary material, Fig. S3). *Campylobacter rectus* is often associated with periodontal disease^5^, ^62^ where it occurs in elevated numbers in the deep periodontal pockets.^63^, ^64^ *Campylobacter* spp. have long been described as of clinical relevance.^65^, ^66^ They are Gram‐negative, microaerophilic bacteria whose motility is conferred by a single polar, glycosylated flagellum.^67^ Even though little has been described about *C. rectus* flagellar glycosylation, the bacterium possesses the genetic make‐up for Pse biosynthesis (V. Friedrich, M. L. Braun, S. Bloch, C. Schäffer, unpublished observation) and, interestingly, also covers its cells with a 2D S‐layer.^67^, ^68^, ^69^

For *P. gingivalis* and *T. forsythia,* a direct synergistic interaction has been described previously, albeit for another strain.^70^ In the biofilms analyzed in this study, *P. gingivalis* OMZ925 seemed to strongly co‐localize with *T. forsythia* ATCC 43037 *∆wecC* (Figure 4B) but was not affected in its growth by the *T. forsythia* cell surface composition (Figure 4). Coaggregation of *P. gingivalis* OMZ925 with *T. forsythia* did not differ significantly between *T. forsythia* wild‐type strains ATCC 43037 and UB4 and their respective mutants and a preferential direct interaction of *P. gingivalis* OMZ925 with *T. forsythia* ATCC 43037 *∆wecC* could not be observed. *Porphyromonas gingivalis* outer membrane vesicles enhance attachment to and invasion of epithelial cells by *T. forsythia*,^71^ co‐infection of *T. forsythia* and *P. gingivalis* increases abscess formation in a mouse model,^72^ and *T. forsythia* cell extracts have been shown to have a growth‐promoting effect on *P. gingivalis*.^73^ In support of the synergistic interaction between the two species, Bao *et al*. described, in the very same experimental model as used here, reduced growth of *T. forsythia* in multispecies biofilms containing a *P. gingivalis* Lys‐gingipain‐deficient strain.^12^ However, despite this observation, the molecular mechanism of coaggregation between the two pathogens is still unclear.^74^

In conclusion, the present study shows that the growth of *T. forsythia* in an *in vitro* multispecies biofilm, as represented by the “Zurich biofilm model”, does not depend on the bacterium's cell surface composition. Deletion of one or more sugars (*T. forsythia* ATCC 43073 *∆pseC*,* ∆wecC, T. forsythia* UB4 *∆legC*) has a disadvantageous effect on neither the biofilm growth of *T. forsythia*, nor on overall cell numbers in the biofilm. *Tannerella forsythia* is able to establish itself in the multispecies consortium even without an S‐layer (*T. forsythia* ATCC 43073 *∆tfsAB)*. These findings suggest that the glycosylated S‐layer of *T. forsythia* does not play a crucial role in regulating the bacterium's growth in a multispecies biofilm. Nevertheless, we observed that it affected the bacterium's localization in the biofilm, the interaction with *C. rectus*, for which the glycosylated S‐layer has a growth retarding effect, and its co‐localization with *P. gingivalis*, which is increased upon a three‐sugar truncation of the O‐glycan in the *T. forsythia* ATCC 43037 *∆wecC* mutant. Hence, changes in the S‐layer and surface glycosylation of *T. forsythia* might actually contribute to the bacterium's virulence potential by promoting structural arrangements within in the biofilm. Whether this contributes to the immune evasion of the biofilm‐associated species needs to be tested in functional interaction assays with host cells.

## CONFLICT OF INTEREST

The authors declare no conflict of interest related to this study.

## References

[1] Marsh PD . Dental plaque: Biological significance of a biofilm and community life‐style. J Clin Periodontol. 2005;32:7‐15. 16128825 10.1111/j.1600-051X.2005.00790.x

[2] Marsh PD . Dental plaque as a microbial biofilm. Caries Res. 2004;38:204‐211. 15153690 10.1159/000077756

[3] Van Dyke TE , Sheilesh D . Risk factors for periodontitis. J Int Acad Periodontol. 2005;7:3‐7. 15736889 PMC1351013

[4] Hajishengallis G , Lamont RJ . Beyond the red complex and into more complexity: The polymicrobial synergy and dysbiosis (PSD) model of periodontal disease etiology. Mol Oral Microbiol. 2012;27:409‐419. 23134607 10.1111/j.2041-1014.2012.00663.x PMC3653317

[5] Socransky SS , Haffajee AD , Cugini MA , Smith C , Kent RL Jr . Microbial complexes in subgingival plaque. J Clin Periodontol. 1998;25:134‐144. 9495612 10.1111/j.1600-051x.1998.tb02419.x

[6] Hajishengallis G . Immunomicrobial pathogenesis of periodontitis: Keystones, pathobionts, and host response. Trends Immunol. 2014;35:3‐11. 24269668 10.1016/j.it.2013.09.001 PMC3947349

[7] Lamont RJ , Jenkinson HF . Life below the gum line: Pathogenic mechanisms of *Porphyromonas gingivalis* . Microbiol Mol Biol Rev. 1998;62:1244‐1263. 9841671 10.1128/mmbr.62.4.1244-1263.1998 PMC98945

[8] Bostanci N , Belibasakis GN . *Porphyromonas gingivalis*: An invasive and evasive opportunistic oral pathogen. FEMS Microbiol Lett. 2012;333:1‐9. 22530835 10.1111/j.1574-6968.2012.02579.x

[9] Kitamura Y , Matono S , Aida Y , Hirofuji T , Maeda K . Gingipains in the culture supernatant of *Porphyromonas gingivalis* cleave CD4 and CD8 on human T cells. J Periodontal Res. 2002;37:464‐468. 12472841 10.1034/j.1600-0765.2002.01364.x

[10] Wingrove JA , DiScipio RG , Chen Z , Potempa J , Travis J , Hugli TE . Activation of complement components C3 and C5 by a cysteine proteinase (gingipain‐1) from *Porphyromonas* (*Bacteroides*) *gingivalis* . J Biol Chem. 1992;267:18902‐18907. 1527018

[11] Lourbakos A , Potempa J , Travis J , et al. Arginine‐specific protease from *Porphyromonas gingivalis* activates protease‐activated receptors on human oral epithelial cells and induces interleukin‐6 secretion. Infect Immun. 2001;69:5121‐5130. 11447194 10.1128/IAI.69.8.5121-5130.2001 PMC98608

[12] Bao K , Belibasakis GN , Thurnheer T , Aduse‐Opoku J , Curtis MA , Bostanci N . Role of *Porphyromonas gingivalis* gingipains in multi‐species biofilm formation. BMC Microbiol. 2014;14:258. 25270662 10.1186/s12866-014-0258-7 PMC4189655

[13] O'Brien‐Simpson NM , Pathirana RD , Walker GD , Reynolds EC . *Porphyromonas gingivalis* RgpA‐Kgp proteinase‐adhesin complexes penetrate gingival tissue and induce proinflammatory cytokines or apoptosis in a concentration‐dependent manner. Infect Immun. 2009;77:1246‐1261. 19114547 10.1128/IAI.01038-08 PMC2643621

[14] Belibasakis GN , Bostanci N , Reddi D . Regulation of protease‐activated receptor‐2 expression in gingival fibroblasts and Jurkat T cells by *Porphyromonas gingivalis* . Cell Biol Int. 2010;34:2872‐2892. 10.1042/CBI20090290 19947912

[15] Dashper SG , Seers CA , Tan KH , Reynolds EC . Virulence factors of the oral spirochete *Treponema denticola* . J Dent Res. 2011;90:691‐703. 20940357 10.1177/0022034510385242 PMC3144123

[16] Lux R , Sim JH , Tsai JP , Shi W . Construction and characterization of a *cheA* mutant of *Treponema denticola* . J Bacteriol. 2002;184:3130‐3134. 12003957 10.1128/JB.184.11.3130-3134.2002 PMC135053

[17] Grenier D , Uitto VJ , McBride BC . Cellular location of a *Treponema denticola* chymotrypsinlike protease and importance of the protease in migration through the basement membrane. Infect Immun. 1990;58:347‐351. 2404867 10.1128/iai.58.2.347-351.1990 PMC258461

[18] Batista da Silva AP , Lee W , Bajenova E , McCulloch CA , Ellen RP . The major outer sheath protein of *Treponema denticola* inhibits the binding step of collagen phagocytosis in fibroblasts. Cell Microbiol. 2004;6:485‐498. 15056218 10.1111/j.1462-5822.2004.00377.x

[19] Amin M , Grove DA , Kapus A , Glogauer M , Ellen RP . An actin‐stabilizing peptide conjugate deduced from the major outer sheath protein of the bacterium *Treponema denticola* . Cell Motil Cytoskeleton. 2007;64:662‐674. 17565753 10.1002/cm.20213

[20] Puthengady TB , Sun CX , Bajenova E , Ellen RP , Glogauer M . Modulation of human neutrophil functions *in vitro* by *Treponema denticola* major outer sheath protein. Infect Immun. 2006;74:1954‐1957. 16495573 10.1128/IAI.74.3.1954-1957.2006 PMC1418645

[21] Miyamoto M , Ishihara K , Okuda K . The *Treponema denticola* surface protease dentilisin degrades interleukin‐1 beta (IL‐1 beta), IL‐6, and tumor necrosis factor alpha. Infect Immun. 2006;74:2462‐2467. 16552080 10.1128/IAI.74.4.2462-2467.2006 PMC1418930

[22] Okuda T , Kimizuka R , Miyamoto M , et al. *Treponema denticola* induces interleukin‐8 and macrophage chemoattractant protein 1 production in human umbilical vein epithelial cells. Microbes Infect. 2007;9:907‐913. 17533151 10.1016/j.micinf.2007.03.009

[23] Sharma A . Virulence mechanisms of *Tannerella forsythia* . Periodontol 2000. 2010;54:106‐116. 20712636 10.1111/j.1600-0757.2009.00332.x PMC2934765

[24] Sabet M , Lee SW , Nauman RK , Sims T , Um H‐S . The surface (S‐) layer is a virulence factor of *Bacteroides forsythus* . Microbiology. 2003;149(Pt 12):3617‐3627. 14663093 10.1099/mic.0.26535-0

[25] Sekot G , Posch G , Oh YJ , et al. Analysis of the cell surface layer ultrastructure of the oral pathogen *Tannerella forsythia* . Arch Microbiol. 2012;194:525‐539. 22273979 10.1007/s00203-012-0792-3 PMC3354324

[26] Posch G , Pabst M , Brecker L , Altmann F , Messner P , Schäffer C . Characterization and scope of S‐layer protein *O*‐glycosylation in *Tannerella forsythia* . J Biol Chem. 2011;286:38714‐38724. 21911490 10.1074/jbc.M111.284893 PMC3207478

[27] Tomek MB , Neumann L , Nimeth I , et al. The S‐layer proteins of *Tannerella forsythia* are secreted via a type IX secretion system that is decoupled from protein *O*‐glycosylation. Mol Oral Microbiol. 2014;29:307‐320. 24943676 10.1111/omi.12062 PMC4232474

[28] Narita Y , Sato K , Yukitake H , et al. Lack of a surface layer in *Tannerella forsythia* mutants deficient in the type IX secretion system. Microbiology. 2014;160(Pt 10):2295‐2303. 25023245 10.1099/mic.0.080192-0 PMC4175972

[29] Dunne WM Jr . Bacterial adhesion: Seen any good biofilms lately? Clin Microbiol Rev. 2002;15:155‐166. 11932228 10.1128/CMR.15.2.155-166.2002 PMC118072

[30] Garrett TR , Bhakoo M , Zhang ZB . Bacterial adhesion and biofilms on surfaces. Prog Nat Sci. 2008;18:1049‐1056.

[31] Nesbitt WE , Doyle RJ , Taylor KG . Hydrophobic interactions and the adherence of *Streptococcus sanguis* to hydroxylapatite. Infect Immun. 1982;38:637‐644. 6292108 10.1128/iai.38.2.637-644.1982 PMC347787

[32] Nesbitt WE , Doyle RJ , Taylor KG , Staat RH , Arnold RR . Positive coooperativity in the binding of *Streptococcus sanguis* to hydroxylapatite. Infect Immun. 1982;35:157‐165. 6172378 10.1128/iai.35.1.157-165.1982 PMC351010

[33] Wu H , Zeng M , Fives‐Taylor P . The glycan moieties and the N‐terminal polypeptide backbone of a fimbria‐associated adhesin, Fap1, play distinct roles in the biofilm development of *Streptococcus parasanguinis* . Infect Immun. 2007;75:2181‐2188. 17296746 10.1128/IAI.01544-06 PMC1865748

[34] Liang X , Chen YY , Ruiz T , Wu H . New cell surface protein involved in biofilm formation by *Streptococcus parasanguinis* . Infect Immun. 2011;79:3239‐3248. 21576336 10.1128/IAI.00029-11 PMC3147580

[35] Guerry P , Ewing CP , Schirm M , et al. Changes in flagellin glycosylation affect *Campylobacter* autoagglutination and virulence. Mol Microbiol. 2006;60:299‐311. 16573682 10.1111/j.1365-2958.2006.05100.x PMC1424674

[36] Guerry P . *Campylobacter* flagella: Not just for motility. Trends Microbiol. 2007;15:456‐461. 17920274 10.1016/j.tim.2007.09.006

[37] Thibault P , Logan SM , Kelly JF , et al. Identification of the carbohydrate moieties and glycosylation motifs in *Campylobacter jejuni* flagellin. J Biol Chem. 2001;276:34862‐34870. 11461915 10.1074/jbc.M104529200

[38] Angata T , Varki A . Chemical diversity in the sialic acids and related alpha‐keto acids: An evolutionary perspective. Chem Rev. 2002;102:439‐469. 11841250 10.1021/cr000407m

[39] Friedrich V , Janesch B , Windwarder M , et al. *Tannerella forsythia* strains display different cell‐surface nonulosonic acids: Biosynthetic pathway characterization and first insight into biological implications. Glycobiology. 2017;27:342‐357. 27986835 10.1093/glycob/cww129 PMC5378307

[40] Varki A . Diversity in the sialic acids. Glycobiology. 1992;2:25‐40. 1550987 10.1093/glycob/2.1.25 PMC7108601

[41] Sakakibara J , Nagano K , Murakami Y , et al. Loss of adherence ability to human gingival epithelial cells in S‐layer protein‐deficient mutants of *Tannerella forsythensis* . Microbiology. 2007;153(Pt 3):866‐876. 17322207 10.1099/mic.0.29275-0

[42] Sekot G , Posch G , Messner P , et al. Potential of the *Tannerella forsythia* S‐layer to delay the immune response. J Dent Res. 2011;90:109‐114. 20929722 10.1177/0022034510384622 PMC4382719

[43] Honma K , Inagaki S , Okuda K , Kuramitsu HK , Sharma A . Role of a *Tannerella forsythia* exopolysaccharide synthesis operon in biofilm development. Microb Pathog. 2007;42:156‐166. 17363213 10.1016/j.micpath.2007.01.003

[44] Guggenheim B , Gmür R , Galicia JC , et al. *In vitro* modeling of host–parasite interactions: The ‘subgingival’ biofilm challenge of primary human epithelial cells. BMC Microbiol. 2009;9:280. 20043840 10.1186/1471-2180-9-280 PMC2818713

[45] Ammann TW , Gmür R , Thurnheer T . Advancement of the 10‐species subgingival Zurich biofilm model by examining different nutritional conditions and defining the structure of the *in vitro* biofilms. BMC Microbiol. 2012;12:227. 23040057 10.1186/1471-2180-12-227 PMC3561252

[46] Ammann TW , Belibasakis GN , Thurnheer T . Impact of early colonizers on *in vitro* subgingival biofilm formation. PLoS ONE. 2013;8:e83090. 24340084 10.1371/journal.pone.0083090 PMC3855599

[47] Ammann TW , Bostanci N , Belibasakis GN , Thurnheer T . Validation of a quantitative real‐time PCR assay and comparison with fluorescence microscopy and selective agar plate counting for species‐specific quantification of an *in vitro* subgingival biofilm model. J Periodontal Res. 2013;48:517‐526. 23278531 10.1111/jre.12034

[48] Belibasakis GN , Thurnheer T . Validation of antibiotic efficacy on *in vitro* subgingival biofilms. J Periodontol. 2014;85:343‐348. 23659420 10.1902/jop.2013.130167

[49] Thurnheer T , Bostanci N , Belibasakis GN . Microbial dynamics during conversion from supragingival to subgingival biofilms in an *in vitro* model. Mol Oral Microbiol. 2016;31:125‐135. 26033167 10.1111/omi.12108

[50] Friedrich V , Pabinger S , Chen T , Messner P , Dewhirst FE , Schäffer C . Draft genome sequence of *Tannerella forsythia* type strain ATCC 43037. Genome Announc. 2015;3:e00660‐00615. 26067981 10.1128/genomeA.00660-15 PMC4463545

[51] Stafford GP , Chaudhuri RR , Haraszthy V , et al. Draft genome sequences of three clinical isolates of *Tannerella forsythia* isolated from subgingival plaque from periodontitis patients in the United States. Genome Announc. 2016;4:e01268‐01316. 27908987 10.1128/genomeA.01286-16 PMC5137401

[52] Posch G , Andrukhov O , Vinogradov E , et al. Structure and immunogenicity of the rough‐type lipopolysaccharide from the periodontal pathogen *Tannerella forsythia* . Clin Vaccine Immunol. 2013;20:945‐953. 23616409 10.1128/CVI.00139-13 PMC3675976

[53] Friedrich V , Gruber C , Nimeth I , et al. Outer membrane vesicles of *Tannerella forsythia*: Biogenesis, composition, and virulence. Mol Oral Microbiol. 2015;30:451‐473. 25953484 10.1111/omi.12104 PMC4604654

[54] Gmür R , Guggenheim B . Antigenic heterogeneity of *Bacteroides intermedius* as recognized by monoclonal antibodies. Infect Immun. 1983;42:459‐470. 6196291 10.1128/iai.42.2.459-470.1983 PMC264452

[55] Thurnheer T , Gmür R , Guggenheim B . Multiplex FISH analysis of a six‐species bacterial biofilm. J Microbiol Methods. 2004;56:37‐47. 14706749 10.1016/j.mimet.2003.09.003

[56] Thurnheer T , van der Ploeg JR , Giertsen E , Guggenheim B . Effects of *Streptococcus mutans gtfC* deficiency on mixed oral biofilms *in vitro* . Caries Res. 2006;40:163‐171. 16508276 10.1159/000091065

[57] Honma K , Mishima E , Inagaki S , Sharma A . The OxyR homologue in *Tannerella forsythia* regulates expression of oxidative stress responses and biofilm formation. Microbiology. 2009;155(Pt 6):1912‐1922. 19389765 10.1099/mic.0.027920-0 PMC2782426

[58] Derrien M , van Passel MW , van de Bovenkamp JH , Schipper RG , de Vos WM , Dekker J . Mucin‐bacterial interactions in the human oral cavity and digestive tract. Gut Microbes. 2010;1:254‐268. 21327032 10.4161/gmic.1.4.12778 PMC3023607

[59] Crouzier T , Jang H , Ahn J , Stocker R , Ribbeck K . Cell patterning with mucin biopolymers. Biomacromol. 2013;14:3010‐3016. 10.1021/bm400447z PMC4076112 23980712

[60] Renner LD , Weibel DB . Physicochemical regulation of biofilm formation. MRS Bull. 2011;36:347‐355. 22125358 10.1557/mrs.2011.65 PMC3224470

[61] Pham TK , Roy S , Noirel J , Douglas I , Wright PC , Stafford GP . A quantitative proteomic analysis of biofilm adaptation by the periodontal pathogen *Tannerella forsythia* . Proteomics. 2010;10:3130‐3141. 20806225 10.1002/pmic.200900448

[62] Macuch PJ , Tanner AC . *Campylobacter* species in health, gingivitis, and periodontitis. J Dent Res. 2000;79:785‐792. 10728981 10.1177/00220345000790021301

[63] Dzink JL , Tanner AC , Haffajee AD , Socransky SS . Gram negative species associated with active destructive periodontal lesions. J Clin Periodontol. 1985;12:648‐659. 3863838 10.1111/j.1600-051x.1985.tb00936.x

[64] Ihara H , Miura T , Kato T , et al. Detection of *Campylobacter rectus* in periodontitis sites by monoclonal antibodies. J Periodontal Res. 2003;38:64‐72. 12558939 10.1034/j.1600-0765.2003.01627.x

[65] Lee S , Lee J , Ha J , et al. Clinical relevance of infections with zoonotic and human oral species of *Campylobacter* . J Microbiol. 2016;54:459‐467. 27350611 10.1007/s12275-016-6254-x

[66] Man SM . The clinical importance of emerging *Campylobacter* species. Nat Rev Gastroenterol Hepatol. 2011;8:669‐685. 22025030 10.1038/nrgastro.2011.191

[67] Bolton DJ . *Campylobacter* virulence and survival factors. Food Microbiol. 2015;48:99‐108. 25790997 10.1016/j.fm.2014.11.017

[68] Dokland T , Olsen I , Farrants G , Johansen BV . Three‐dimensional structure of the surface layer of *Wolinella recta* . Oral Microbiol Immunol. 1990;5:162‐165. 2080071 10.1111/j.1399-302x.1990.tb00415.x

[69] Thompson SA . *Campylobacter* surface‐layers (S‐layers) and immune evasion. Ann Periodontol. 2002;7:43‐53. 16013216 10.1902/annals.2002.7.1.43 PMC2763180

[70] Yao ES , Lamont RJ , Leu SP , Weinberg A . Interbacterial binding among strains of pathogenic and commensal oral bacterial species. Oral Microbiol Immunol. 1996;11:35‐41. 8604253 10.1111/j.1399-302x.1996.tb00334.x

[71] Inagaki S , Onishi S , Kuramitsu HK , Sharma A . *Porphyromonas gingivalis vesicles* enhance attachment, and the leucine‐rich repeat BspA protein is required for invasion of epithelial cells by *Tannerella forsythia* . Infect Immun. 2006;74:5023‐5028. 16926393 10.1128/IAI.00062-06 PMC1594857

[72] Yoneda M , Hirofuji T , Anan H , et al. Mixed infection of *Porphyromonas gingivalis* and *Bacteroides forsythus* in a murine abscess model: Involvement of gingipains in a synergistic effect. J Periodontal Res. 2001;36:237‐243. 11519697 10.1034/j.1600-0765.2001.036004237.x

[73] Yoneda M , Yoshikane T , Motooka N , et al. Stimulation of growth of *Porphyromonas gingivalis* by cell extracts from *Tannerella forsythia* . J Periodontal Res. 2005;40:105‐109. 15733144 10.1111/j.1600-0765.2005.00774.x

[74] Zhu WD , Lee SW . Surface interactions between two of the main periodontal pathogens: *Porphyromonas gingivalis* and *Tannerella forsythia* . J Periodontal Implant Sci. 2016;46:2‐9. 26937289 10.5051/jpis.2016.46.1.2 PMC4771834

[75] Züger J , Lüthi‐Schaller H , Gmür R . Uncultivated *Tannerella* BU045 and BU063 are slim segmented filamentous rods of high prevalence but low abundance in inflammatory disease‐associated dental plaques. Microbiology. 2007;153(Pt 11):3809‐3816. 17975090 10.1099/mic.0.2007/010926-0

[76] Zijnge V , van Leeuwen MB , Degener JE , et al. Oral biofilm architecture on natural teeth. PLoS ONE. 2010;5:e9321. 20195365 10.1371/journal.pone.0009321 PMC2827546

