# Supplementary material for: Behavior of two Tannerella forsythia strains and their cell surface mutants in multispecies oral biofilms
Source: Mol Oral Microbiol. 2017 May 22;32(5):404–18. doi: 10.1111/omi.12182 (PMC5600126; doi:10.1111/omi.12182)
Supplement: Supplementary file 1 [file OMI-32-404-s001.pdf]

Supporting Information

Behavior of two *Tannerella forsythia* strains and their cell surface mutants in multispecies oral biofilms

Susanne Bloch, Thomas Thurnheer, Yukitaka Murakami, Georgios N. Belibasakis, Christina Schäffer

SUPPORTING MATERIAL AND METHODS

Planktonic growth of *T. forsythia*

In order to evaluate the growth kinetics of both *T. forsythia* ATCC 43037 and UB4 wild-type strains as well as their respective mutants, bacteria were grown for 3 days in BHI supplemented with *N*-acetylmuramic acid, horse serum and 50 µg/ml gentamycin. From these cultures, 10 ml of fresh BHI was inoculated at a final OD_{600} of 0,05. The OD_{600} was measured in 24 hour intervals for 8 d [1]. Growth curves were obtained from three individual experiments with two technical replicates each.

Coaggregation assay

Coaggregation assays were performed according to Shimotahira *et al.* [1]. In brief, *T. forsythia* wild-type and mutant strains were grown in BHI supplemented with *N*-acetyl-muramic acid, horse serum and gentamycin. *Porphyromonas gingivalis* (OMZ925) was cultivated in BHI (without supplements), *C. rectus* OMZ388 in mFUM supplemented with 0.1% NaFF. All strains were harvested by centrifugation and resuspended in coaggregation buffer (1 mM $CaCl_2$, 1 mM $MgCl_2$, 150 mM NaCl, and Tris-HCl [pH 8.0]). The suspensions were adjusted to an OD_{600} of 0.5. Coaggregation partner strains were mixed in a 1:1 ratio in a disposable cuvette. Additionally, for each strain the autoaggregation was determined by measuring single bacterial cells only. Suspensions were incubated at room temperature and were measured at 15-min intervals using a spectrophotometer. Decreases in absorbance indicated cell aggregates precipitating to the bottom of the cuvette. The percentage of aggregation (AI) was calculated as: $\mathrm{AI} = \frac{(\text{initial OD600} - \text{post incubation OD600})}{\text{initial OD600}} \text{ x } 100$. Data represent mean values ± SD of five independent experiments with three replicates each for coagggregation *P. gingivalis* and four independent experiments with three replicates each for coagggregation with *C. rectus*. Statistical analysis was performed using the unpaired Student's t-Test. Asterisks indicate significant differences (*, $P < 0.05$).

SUPPORTING TABLES

Table S1. Doubling times of *T. forsythia* wild-type and mutant strains used in this study. Doubling times (mean values ±SD) are shown. Significant differences between strains were determined by the unpaired Student's t-Test (*P≤0.05*); *significantly higher *versus* *T. forsythia* ATCC 43037 wild-type and complemented strain Δ$pseC_{comp}$, ¶ significantly higher *versus T. forsythia* ATCC 43037 wild-type, # significantly higher *versus T. forsythia* ATCC 43037 Δ*wecC.*

***T. forsythia* strain**	**Doubling time (h) ±SD**	**Significance**
ATCC43037 WT	14.41 ±1.41	
ATCC43037 Δ*pseC*	25.59 ±9.29	*#
ATCC43037 Δ*wecC*	17.80 ±2.38	¶
ATCC43037 Δ*tfsAB*	20.74 ±5.76	*
ATCC43037 Δ$pseC_{comp}$	14.99 ±3.64	
UB4 WT	16.75 ±9.58	
UB4 Δ*legC*	19.07 ±8.40	
UB4 Δ$legC_{comp}$	12.28 ±3.75	

SUPPORTING FIGURES

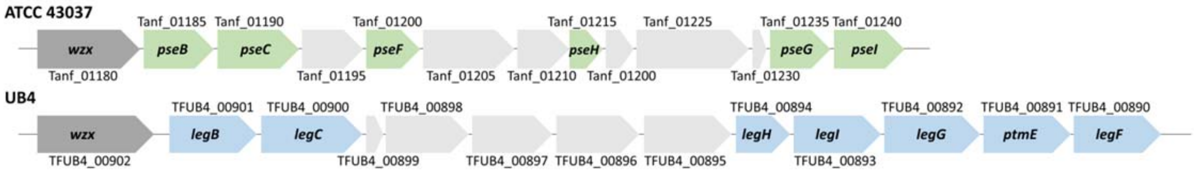

Figure S1. Nonulosonic acid biosynthesis locus of *T. forsythia* ATCC 43037 and *T. forsythia* UB4 wild-type strains. Pse biosynthesis genes (*pseB*, *pseC*, *pseH*, *pseG*, *pseI* and *pseF*) present in the type strain ATCC 43037 are shown in red. Corresponding genes for Leg biosynthesis (*legB*, *legC*, *legH*, *legI*, *legG*, *legF* and a gene encoding a predicted nucleotidyl transferase (*ptmE*) are shown in green for the UB4 strain [2]. NCBI locus tags are shown for each gene.

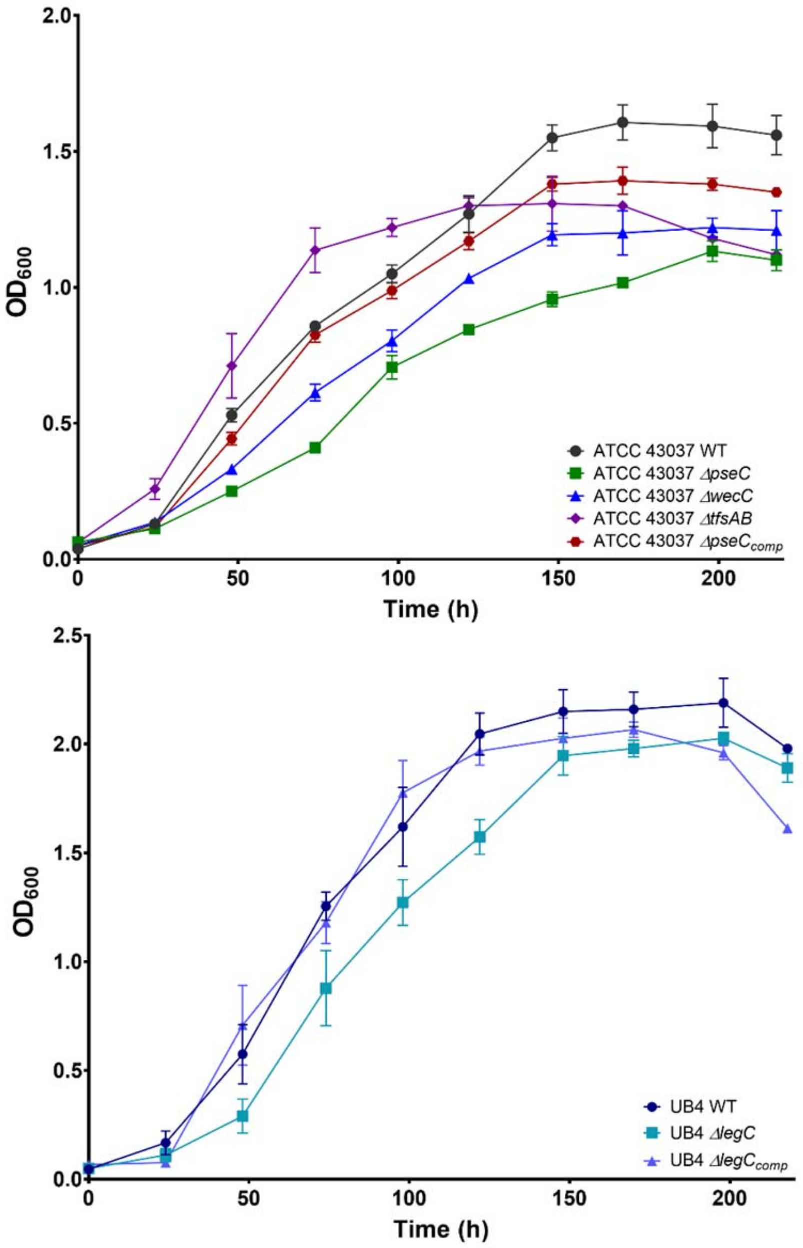

Figure S2. Growth curves of *T. forsythia* wild-type strains and mutants included in this study. Growth of *T. forsythia* strains was monitored by measuring the OD_{600} at 24-hour intervals in three independent experiments with two technical replicates per strain. Data are presented as mean ±SEM. **(A)** Growth curves of ATCC 43037 wild-type, ATCC 43037 Δ*pseC*, Δ*wecC,* Δ*tfsAB* mutants, and complemented strain Δ$pseC_{comp}$ and **(B)** *Tannerella forsythia* UB4 wild-type, UB4 Δ*legC* mutant and complemented strain Δ$legC_{comp}$.

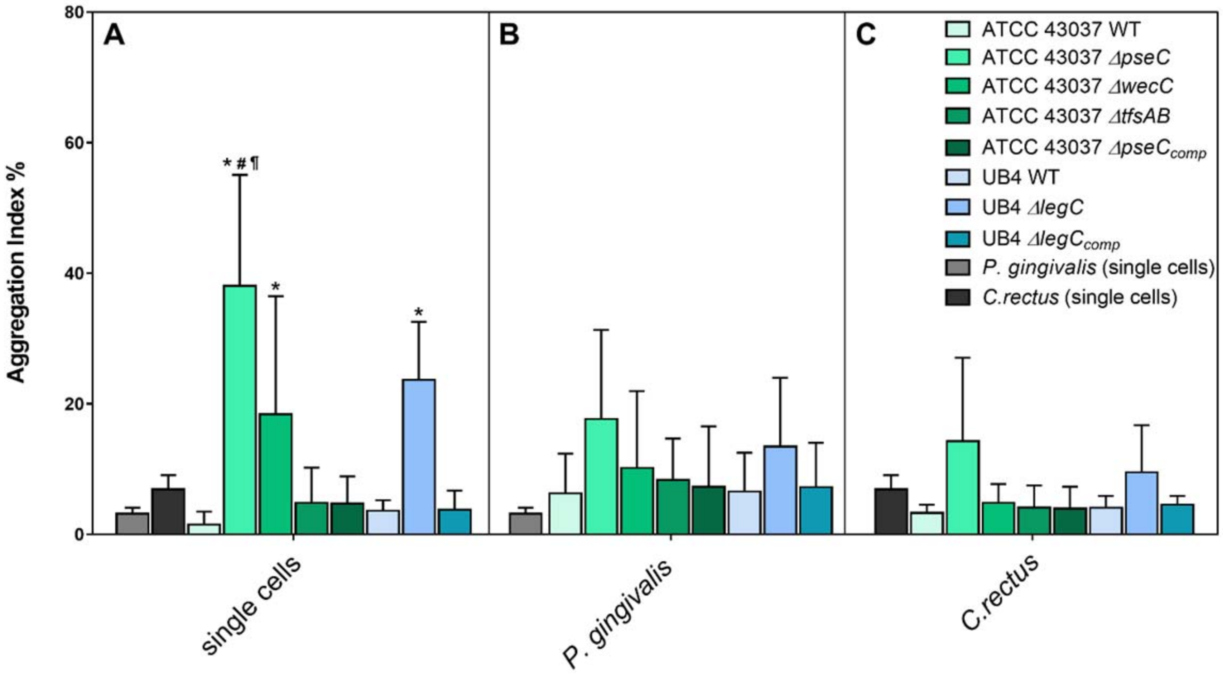

Figure S3. Aggregation of *T. forsythia* wild-type strains and mutants with *P. gingivalis* and *C. rectus*. Each *T. forsythia* strain was mixed with its co-aggregation partner in a 1:1-ratio and the OD_{600} of the suspension was measured in 15-minute intervals. For each strain,

the autoaggregation (AI) was calculated as: $\mathrm{AI} = \frac{(\text{initial OD600} - \text{post incubation OD600})}{\text{initial OD600}} \text{ x } 100$.

The coaggregation rate (CR) was determined as: $\mathrm{CR} = \frac{(\mathrm{AIx+AIy})}{2} - \frac{\mathrm{AI.x+y}}{(\mathrm{AIx+AIy})/2},$, where AI_x and AI_y represent the aggregation of one species alone and AI_{x+y} represents the aggregation of the mixture of *T. forsythia* with *P. gingivalis* or *C. rectus*, respectively [3]. Mean values ±SD of five independent experiments for coagggregation with *P. gingivalis* and four independent experiments for coagggregation with *C. rectus* are shown. Statistical significance was tested by ANOVA (Tukey's post-hoc test for multiple comparisons, P≤0.05) using GraphPad Prism version 7.00 for Windows (GraphPad Software, La Jolla California USA); * significantly higher *versus* parental wild-type and complemented strain, ¶ significantly higher *versus T. forsythia* ATCC 43037 Δ*tfsAB*, # significantly higher *versus T. forsythia* ATCC 43037 Δ*wecC*.

References

1. Shimotahira, N, Oogai, Y, Kawada-Matsuo, M, et al. The surface layer of *Tannerella forsythia* contributes to serum resistance and oral bacterial coaggregation. *Infect Immun.* 2013;81(4):1198-1206.
2. Friedrich, V, Janesch, B, Windwarder, M, et al. *Tannerella forsythia* strains display different cell-surface nonulosonic acids: biosynthetic pathway characterization and first insight into biological implications. *Glycobiology.* 2017;doi:10.1093/glycob/cww129.
3. Cheng, Z, Meng, X, Wang, H, Chen, M, Li, M. Isolation and characterization of broad spectrum coaggregating bacteria from different water systems for potential use in bioaugmentation. *PLoS One.* 2014;9(4):e94220.
